# A comprehensive cross-sectional study on the current status of proactive health awareness among Guangxi healthcare workers and its influencing factors: understanding from both individual and variable viewpoints

**DOI:** 10.3389/fpubh.2025.1588640

**Published:** 2025-09-11

**Authors:** Wuzhao Chen, Qinyi Guan

**Affiliations:** ^1^Institute of Hospital Management and Medical Prevention Collaborative Innovation, Guangxi Academy of Medical Sciences, Nanning, China; ^2^The Fifth People’s Hospital of Nanning, Nanning, China

**Keywords:** proactive health, latent class model, healthcare institution staff, influencing factors, cross-sectional study

## Abstract

**Background:**

Enhancing awareness of proactive health concepts among healthcare professionals is vital in the context of an aging population and limited resources, shifting the focus from “patients and diseases” to “individuals and health.”

**Methods:**

Using a self-developed Health Action Survey Questionnaire, this study assessed the proactive health cognition of randomly selected healthcare providers in Guangxi Zhuang Autonomous Region. Analyses included latent class models, binary logistic regression, and mediation effects.

**Results:**

Among 173,892 participants, 77,307 (44.46%) were unaware of proactive health. Significant differences in health behaviors cognition and willingness to disseminate health knowledge identified 4 groups among healthcare staff. Those with low health action cognition were more likely to know about proactive health, while those with high cognition and willingness had lower awareness. Health action cognition and willingness also indirectly influenced awareness through satisfaction with government health initiatives.

**Conclusion:**

Variations in health action cognition and willingness among Guangxi healthcare staff impact their awareness of proactive health. Relevant departments should prioritize healthcare workers who demonstrate both high awareness of the “Healthy China Action (2019–2030)” and the “Healthy Guangxi 2030” plan and a strong willingness to engage in health-science popularization, focusing on enhancing their satisfaction with government health initiatives to improve public-health outcomes.

## 1 Introduction

In recent years, China has implemented a series of policies to effectively improve the national health status, including the “Healthy China 2030 Planning Outline” and the “State Council’s Opinions on Implementing the Healthy China Action” (1, 2). In its 2019 guidelines, the State Council explicitly emphasized the need to accelerate the transition from focusing on disease treatment to improving public health, advocating for a prevention-oriented approach across all sectors of society (2, 3). As the Healthy China strategy advances, the concept of “proactive health” has gained increasing recognition. Proactive health is centered on people’s health, integrating and coordinating the advantageous resources of multiple fields, disciplines, and institutions, with the aim of achieving a higher level of public health (4–6). It is noteworthy that proactive health emphasizes stimulating individuals’ pursuit of health and their ability to manage their own health, while enhancing individual health awareness (7). As a comprehensive medical model, proactive health is led by health administration departments, with policy guidance, resource sharing, and service integration, encouraging public participation (5). Through interventions in lifestyle factors such as exercise and nutrition, proactive health aims to improve individual health literacy, reduce the risk of disease, and ultimately create an environment where the whole population is engaged, collaborates, and continuously participates, thereby promoting the realization of overall health (6, 7). In this process, individuals are seen as the ‘primary responsible person’ for their own health, both the implementer and the beneficiary (6).

The traditional healthcare service model focuses on the treatment of diseases and patients, but the neglecting the prevention and management of health and risk factors were underestimated. Contemporary Chinese society is encountering the massive pressures from population aging and medical resource limitation (8–11). It’s indicated that by 2025, the demand for nurses is expected to increase by approximately 20% across all countries, underscoring the importance of optimizing nursing workforce planning as a crucial factor in reducing health inequalities and ensuring sustainable healthcare systems (12). The United States will need 20,000 primary care providers at minimum, particularly in rural and resource-limited areas. The recruitment and retention of healthcare professionals will encounter even greater challenges in these regions (13). In comparison, China will be in a more severe aging situation, with large population and a significant number of residents in rural areas, resulting in an urgent demand for healthcare personnel.”

To cope with these challenges, it is essential to improve healthcare staff’s understanding of proactive health concepts. This transformation will help change the service philosophy from being ‘patient and disease-centered’ to one that is ‘people and health-centered’ (14). This change not only promotes a shift from intervening in the causes of illness to addressing risk factors but also enables more effective disease prevention, reduces costs, and enhances the accessibility of healthcare services (15, 16). Therefore, understanding healthcare personnel’s awareness of proactive health is crucial for improving public health levels. However, the level of healthcare workers’ awareness of healthy behaviors and their willingness to participate in health promotion activities often affects their understanding of proactive health. If healthcare staff lack a deep understanding of health actions or show little enthusiasm for participating in health education initiatives, this may limit the effective implementation of proactive health concepts (17). Additionally, previous research on ‘healthcare workers’ has primarily concentrated on specific groups, such as frontline clinical staff, primary healthcare providers, or personnel from specialized public health institutions. Research on healthcare administrators who are responsible for formulating and overseeing medical policies is relatively limited. In fact, all of the aforementioned groups are integral components of the healthcare workforce, and their awareness of healthy behaviors, along with their willingness to participate in health promotion activities, has a direct impact on the quality and effectiveness of healthcare services. Therefore, this study focuses on the impact of healthcare workers’ awareness of healthy behaviors, along with their willingness to engage in health promotion activities, within hospitals, primary healthcare institutions, and specialized public health organizations, on their understanding of proactive health.

The variable-centered research approach is a widely adopted method in the social sciences, aimed at exploring the relationships between variables (18). In contrast, the individual-centered research approach relaxes this assumption by considering that the sample consists of subgroups with different characteristics (18). Therefore, identifying these subgroups and exploring their relationships with predictive and outcome factors becomes an important objective of the research. Currently, research on healthcare personnel’s awareness of health actions and their willingness to engage in health promotion is typically assessed using overall scores from scales, without taking into account individual differences in cognitive performance. Latent Class Analysis (LCA) can effectively classify healthcare personnel with differing levels of awareness of health actions and willingness to engage in health promotion into several subgroups. This approach allows for the exploration of heterogeneity within the population and provides specific support for understanding how different groups acquire knowledge about proactive health (19, 20).

In summary, this study aims to explore the level of awareness regarding proactive health knowledge among personnel in medical institutions in Guangxi. By identifying health action perceptions and the willingness to engage in health education across different subgroups, this research seeks to provide empirical support for the awareness rate of proactive health among staff in Guangxi’s medical institutions, thereby promoting public health.

## 2 Methods

### 2.1 Participants

The Guangxi Zhuang Autonomous Region is one of the provinces in China with the highest number of ethnic minorities. This cross-sectional study was conducted in Guangxi from April 1 to April 30, 2023, using the online platform ‘Wenjuanxing’ ^1^to generate a QR code for the survey, which medical institution staff scanned to participate in the survey. With the support of the Guangxi health administration department, the research team distributed survey notices to various medical institutions (including hospitals, public health institutions, and primary healthcare centers) through the health committees of 14 cities in Guangxi. These institutions were instructed to provide medical staff with detailed explanations of the survey’s significance and content. Only after obtaining the consent of the medical staff were they informed of the instructions for completing the questionnaire and provided with the QR code for this study, which they would scan to participate in the survey. Each participant signed an informed consent form. The survey was conducted anonymously, and the system automatically generated a unique ID for each completed questionnaire. To prevent duplicate submissions, the system restricts each IP address and device to one submission only. The platform also records the location and the time spent on completing the questionnaire.

The inclusion criteria for the study subjects were as follows: (1) aged over 18 years; (2) registered to practice and working in Guangxi; (3) formally employed; (4) willing to participate in the survey. A total of 177,625 subjects were included in this study, with the exclusion criteria comprising: (1) logical errors (*N* = 936); (2) missing information (*N* = 291); (3) organization type classified as other institutions (*N* = 2,536). Ultimately, 173,862 subjects were included in the analysis. In 2022, Guangxi’s healthcare institutions had a total workforce of 517,100, as reported in the Guangxi Health Care Development Statistics Bulletin (21). The effective sample obtained in this survey represented 33.62% of this population. This study has been approved by the Ethics Committee of the People’s Hospital of Guangxi Zhuang Autonomous Region (Ethics No: KY-IIT-2024-141). All methods are carried out in accordance with the relevant guidelines and regulations.

### 2.2 Survey instrument

The “Health Action Survey Questionnaire” consists of three sections: basic demographic information, health action cognition, and willingness to engage in health education. This questionnaire aims to systematically assess respondents’ awareness of national and local health policies, as well as their views on participating in health promotion activities. The section of the questionnaire concerning health action cognition and willingness to engage in health education comprises nine items, scored using a five-point Likert scale. The scores range from 1 (indicating “very knowledgeable”) to 5 (indicating “not at all knowledgeable”). A higher score reflects a lower level of awareness regarding health actions and a reduced willingness to participate in health promotion activities. Items 1 and 2 primarily assess respondents’ awareness of two key health policies: the “Healthy China Action (2019–2030)” and the “Health Guangxi 2030″ plan. The response options range from “very knowledgeable” to “not at all knowledgeable.” Items 3 to 5 focus on evaluating respondents’ perceptions of the importance of health education in promoting public health and enhancing the professional value of healthcare personnel. A rating scale ranging from “very important” to “not important at all” is employed. Items 6 and 7 explore respondents’ enthusiasm for participating in health education activities and their willingness to acquire new knowledge and skills to enhance the effectiveness of health education. The response options range from “very willing” to “not willing at all.” Finally, Items 8 and 9 assess respondents’ self-perception of their health knowledge and their ability to grasp cutting-edge information, using a rating scale ranging from “strongly agree” to “strongly disagree.” The Cronbach’s alpha coefficient for this questionnaire is 0.892, indicating good internal consistency.

### 2.3 Covariate

This study collected general sociodemographic information from healthcare providers and adjusted for it as a covariate. The covariates include gender (male/female), ethnicity (Han/Zhuang/other), marital status (unmarried/married/other), and age (18 to 35 years/36 to 60 years/over 60 years). Social factors comprise years of service (5 years or less/6 to 10 years/11 to 20 years/more than 20 years), type of institution (hospital/primary healthcare facility/specialized public health institution), highest level of education (college and below/bachelor’s/master’s/doctorate), professional technical title (no title/junior title/intermediate title/associate senior title/senior title), and self-rated health status (good/fair/poor).

### 2.4 Statistical method

This study employed SPSS (version 26.0), Mplus (version 7.2), and R language (version 4.2.0) for the statistical analysis. Mplus was used to establish a latent class model for health action awareness and willingness to engage in health education among healthcare workers in Guangxi, with analyses conducted across 1 to 6 categories. The optimal model was selected based on the fit indices. The latent class fit indices included the Akaike Information Criterion (AIC), the Bayesian Information Criterion (BIC), and the adjusted BIC (aBIC). Smaller statistical values indicate a better model fit, and the model with the smallest BIC is generally selected as the best model. The entropy value is used to assess classification, with a value of ≥ 0.8 indicating that the model is acceptable. The Lo–Mendell–Rubin likelihood ratio (LMR) and the Bootstrap likelihood ratio test (BLRT) are used to compare the fit differences between latent profile models; if the *p*-value reaches significance, it indicates that the model with k categories is significantly better than the model with k-1 categories. Subsequently, SPSS 26.0 was used for descriptive statistics and χ^2^ tests, and binary logistic regression analysis was conducted to evaluate the influencing factors of proactive health awareness. Finally, to further explore the impact of health action awareness and willingness to promote science among healthcare staff in Guangxi on their proactive health awareness, this study quantitatively assessed health action awareness and willingness to promote science based on cumulative scores. A new mediating variable—satisfaction with current government health action measures—was introduced, centered around these variables. Based on these quantitative variables, a mediating effect model was constructed to reveal the underlying mechanisms between health action awareness, willingness to promote science, and proactive health awareness. The significance level for all statistical tests was set at *α* = 0.05.

## 3 Results

### 3.1 Basic information

This study employed SPSS (version 26.0), Mplus (version 7.2), and R language (version 4.2.0) for the statistical analysis. Mplus was used to establish a latent class model for health action awareness and willingness to engage in health education among healthcare workers in Guangxi, with analyses conducted across 1 to 6 categories.

### 3.2 Results of latent class analysis

Table 1 presents the results of the latent class analysis. The results indicate that as the number of potential categories increases, the values of the AIC, BIC, and aBIC information criteria gradually decrease, suggesting an improvement in the model’s fit. At the same time, entropy, as a measure of the model’s classification accuracy, gradually increases across the models with two to four categories, indicating better classification performance. In Class 4, not only do the information criteria reach their lowest values, but entropy also achieves its highest value of 0.917, indicating the best classification performance. Additionally, the *p*-values of the Lo–Mendell–Rubin test (pLMR) and the Bootstrap Likelihood Ratio Test (pBLRT) for the models with two to four categories are all less than 0.05, suggesting statistical significance and further supporting the validity of the model classification. However, when the model increased to 5 and 6 categories, the information criteria continue to decrease, the increase in entropy shows a slowing trend, and the p-value of the pLMR is no longer statistically significant, indicating that further increasing the number of categories does not significantly improve the classification performance of the model. Therefore, considering the model’s goodness of fit, classification performance, and statistical significance, the four-category model is the optimal choice.

**Table 1:** Model fit metrics for potential profiles of health action awareness and science popularization willingness.

Class	K	AIC	BIC	aBIC	Entropy	pLMR	pBLRT	Class probability (%)
1	35	3119718.073	3120070.390	3119959.158	-	-	-	-
2	71	2556672.603	2557387.302	2557161.661	0.927	<0.001	<0.001	38.73/61.27
3	107	2418364.661	2419441.743	2419101.693	0.888	<0.001	<0.001	28.73/40.76/30.51
4	143	2303555.500	2304994.965	2304540.505	0.917	<0.001	<0.001	24.40/10.99/28.39/36.22
5	179	2223031.342	2224833.190	2224264.320	0.925	0.718	<0.001	21.27/22.49/27.20/10.75/18.29
6	215	2176591.832	2178756.063	2178072.784	0.926	0.745	<0.001	15.90/18.25/20.96/13.77/22.91/8.21

AIC, Akaike information criterion; BIC, Bayesian information criterion; aBIC, adjusted BIC; pLMR, *p*-value for Lo Mendell–Rubin adjusted likelihood ratio test for K vs. K - 1 profiles; pBLRT = *p*-value for bootstrapped likelihood ratio test.

**Figure 1:**
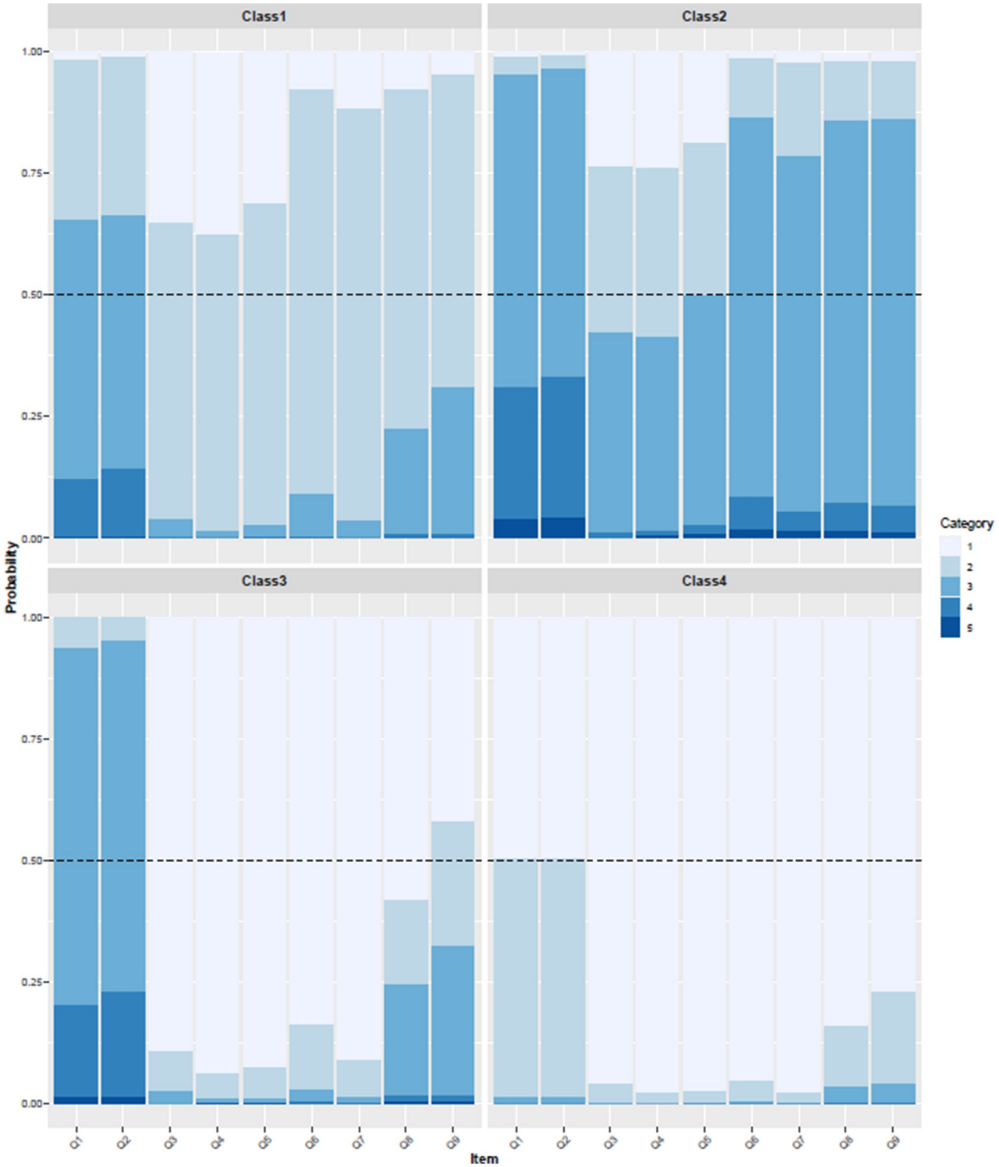
Stacked bar chart of LCA classification results. Attention: (1) Q1: Your understanding of the “Healthy China Action (2019–2030)”; Q2: Your understanding of the “Health Guangxi 2030 Plan”; Q3: The importance of health education initiatives; Q4: The importance of health education for promoting public health; Q5: The importance of health education for enhancing the professional value of healthcare workers; Q6: Are you willing to engage in health education initiatives?; Q7: Are you willing to acquire new knowledge and skills to improve health education activities?; Q8: You possess sufficient health knowledge to meet the demands of health education work; Q9: You can promptly keep up with the latest knowledge in your field; (2) Categories 1–5 represent a scale from very knowledgeable (agree/willing) to very unknowledgeable (disagree/unwilling); (3) Class 1: General Health Action Awareness and General Science Popularization Willingness; Class 2: Low Health Action Awareness and Low Science Popularization Willingness; Class 3: Low Health Action Awareness and High Science Popularization Willingness; Class 4: High Health Action Awareness and Positive Science Popularization Willingness.

On this basis, this study further obtained the conditional probability plots for the four categories of health action awareness and science popularization willingness among staff in Guangxi medical institutions across nine observable indicators, as shown in Figure 1. Class 1: The probability that this group has a general understanding of health action plans exceeds 50%. Although the probabilities for the other seven science popularization-related items also exceed 50% and are considered relatively important/willing, their responses regarding these items are only generally positive compared to the other three classes. Therefore, this class was classified into General Health Action Awareness and General Science Popularization Willingness. Class 2: This group has a relatively limited understanding of the “Healthy China Action (2019–2030)” and the “Healthy Guangxi 2030” plan (with a general probability > 75%), and their recognition and willingness to participate in health science popularization work are low (with four items having a general probability > 75%). Therefore, this class is stratified into Low Health Action Awareness and Low Science Popularization Willingness. Class 3: This group has a low level of understanding of health action plans; however, compared to Classes 1 and 2, their willingness to participate in health science popularization work is very high. Therefore, this class is assigned as Low Health Action Awareness and High Science Popularization Willingness. Class 4: This group has a high level of awareness regarding the “Healthy China Action (2019–2030)” and the “Healthy Guangxi 2030” plan, and they demonstrate a very strong willingness to participate in health science popularization work (with nine items rated as very understood/important/willingness having a probability > 50%). Therefore, this class is named High Health Action Awareness and Positive Science Popularization Willingness.

### 3.3 Awareness of proactive health among different demographic groups

The results demonstrate statistically significant differences (*p* < 0.05) across various demographic and experiential factors, including ethnicity, age, marital status, years of employment, the nature of the employing organization, educational attainment, professional title, health status, perceptions of doctor-patient relationships, experience in creating popular science materials, prior involvement in science popularization activities, membership in the organization’s expert database for science popularization, and proactive health awareness as reflected in the profile. Detailed findings are presented in Table 2.

**Table 2:** Comparison of awareness of proactive health among healthcare institution personnel with different characteristics.

Variable	Know number (%)	Unknown number (%)	Total number (%)	*χ^2^*	*p*
Gender				0.442	<0.506
Male	24,510 (25.38)	19,510 (25.24)	44,020 (25.31)		
Female	72,075 (74.62)	57,797 (74.76)	129,872 (74.69)		
Nation				28.809	<0.001
Han	56,304 (58.29)	44,500 (57.56)	100,804 (57.97)		
Zhuang	32,934 (34.10)	26,404 (34.15)	59,338 (34.12)		
Other	7,347 (7.61)	6,403 (8.28)	13,750 (7.91)		
Marriage				74.476	<0.001
Unmarried	24,286 (25.14)	20,850 (26.97)	45,136 (25.96)		
Married	69,283 (71.73)	54,107 (69.99)	123,390 (70.96)		
Other	3,016 (3.12)	2,350 (3.04)	5,366 (3.09)		
Age (years)				53.260	<0.001
18–35	52,372 (54.22)	43,185 (55.86)	95,557 (54.95)		
36–60	43,863 (45.51)	33,906 (43.86)	77,769 (44.72)		
>60	350 (0.36)	216 (0.28)	566 (0.33)		
Work experience (years)				160.668	<0.001
≤5	24,728 (25.60)	20,720 (26.80)	45,448 (26.14)		
6–10	25,144 (26.03)	20,654 (26.72)	45,798 (26.34)		
11–20	27,138 (28.10)	22,099 (28.59)	49,237 (28.31)		
>20	19,575 (20.27)	13,834 (17.89)	33,409 (19.21)		
Type of institution				15.799	<0.001
Hospital	73,622 (76.23)	54,959 (76.91)	133,081 (76.53)		
Primary healthcare institution	20,213 (20.93)	15,832 (20.48)	36,045 (20.73)		
Specialized public health institution	2,750 (2.85)	2016 (2.61)	4,766 (2.74)		
Education				45.471	<0.001
Associate degree or Below	38,924 (40.30)	30,262 (39.15)	69,186 (39.79)		
Bachelor’s degree	53,132 (55.01)	43,397 (56.14)	96,529 (55.51)		
Master’s degree	4,084 (4.23)	3,394 (4.39)	7,478 (4.40)		
Doctoral degree	445 (0.46)	254 (0.33)	699 (0.40)		
Professional technical title				277.598	<0.001
No title	18,803 (19.47)	15,011 (19.42)	33,814 (19.45)		
Junior title	39,787 (41.19)	32,093 (41.51)	71,880 (41.34)		
Intermediate title	24,556 (25.42)	21,123 (27.32)	45,679 (26.27)		
Associate senior title	11,801 (12.22)	8,278 (10.71)	20,079 (11.55)		
Senior title	1,638 (1.70)	802 (1.04)	2,440 (1.40)		
Health Status				2282.630	<0.001
Good	75,849 (78.53)	53,109 (68.70)	128,958 (74.16)		
Average	19,309 (19.99)	21,849 (28.26)	41,158 (23.67)		
Below average	1,427 (1.48)	2,349 (3.04)	3,776 (2.17)		
Popular science publications				7149.801	<0.001
Yes	21,009 (21.75)	5,480 (7.09)	26,489 (15.23)		
No	75,576 (78.25)	71,827 (92.91)	147,403 (84.77)		
Health education and promotion activities				15149.933	<0.001
Yes	59,705 (61.82)	24,837 (32.13)	84,542 (48.62)		
No	36,880 (38.18)	52,470 (67.87)	89,350 (51.38)		
Doctor-patient relationship				11978.536	<0.001
Harmonious	61,736 (63.92)	29,372 (37.99)	91,108 (52.39)		
Moderate	29,794 (30.85)	38,364 (49.63)	68,158 (39.20)		
Conflictual	5,055 (5.23)	9,571 (12.38)	14,626 (8.41)		
Science popularization experts				10251.351	<0.001
Yes	21,636 (22.40)	3,939 (5.10)	25,575 (14.71)		
No	74,949 (77.60)	73,368 (94.90)	148,317 (85.29)		
Profile				20943.098	<0.001
Class 1	19,723 (20.42)	22,700 (29.36)	42,423 (24.40)		
Class 2	5,737 (5.94)	13,371 (17.30)	19,108 (10.99)		
Class 3	22,286 (23.07)	27,082 (35.03)	49,368 (28.39)		
Class 4	48,839 (50.57)	14,154 (18.31)	62,993 (36.23)		

### 3.4 Results of binary logistic regression

This study first employed binary logistic regression analysis, using proactive health awareness as the dependent variable, to evaluate the relationships between significant variables identified in the univariate analysis and levels of awareness. Subsequently, these significant variables were incorporated into a multifactorial model for adjustment, allowing for a more in-depth analysis of their independent associations with proactive health awareness. Detailed results can be found in Table 3. Compared to the Han ethnic group, individuals from other ethnic minorities, excluding the Zhuang, were at a higher risk of not having heard of proactive health measures [R_OR_: 1.037 (1.064, 1.143), *p* < 0.001]. A comprehensive cross-sectional study on the current situation of proactive health awareness among Guangxi healthcare workers and its contributing factors provides insight from both individual and variable viewpoints. However, after adjusting for covariates in the model, this relationship dissipated. Compared to the unmarried population, both married individuals and those in other marital statuses were reported to perceive lower risks associated with proactive health. However, in Model 2, these relationships disappeared. Compared to the 18–35 age group, individuals aged 36–60 [R_OR_: 0.937 (0.920, 0.955), *p* < 0.001] and those over 60 [R_OR_: 0.748 (0.631, 0.887), *p* < 0.001] exhibited a lower risk of being unaware of proactive health. However, in Model 2, this relationship for the 36–60 age group changed, indicating that individuals aged 36–60 who are unaware of proactive health face a higher risk [A_OR_: 1.079 (1.042, 1.117), *p* < 0.001], while this relationship disappears from those over 60. Regardless of whether in Model 1 or Model 2, individuals with more than 20 years of work experience exhibit a lower risk of being unaware of proactive health compared to those with less than 6 years of experience [Model 1 R_OR_: 0.843 (0.820, 0.868), *p* < 0.001; Model 2 A_OR_: 0.908 (0.861, 0.957), *p* < 0.001]. Compared to hospitals, primary healthcare institutions [R_OR_: 0.970 (0.947, 0.993), *p* < 0.001] and specialized health institutions [R_OR_: 0.908 (0.856, 0.962), *p* < 0.001] exhibit a lower risk of being unaware of proactive health; however, this relationship disappears in Model 2. In contrast, individuals who have received undergraduate education [R_OR_: 1.186 (1.152, 1.220), *p* < 0.001], postgraduate education [R_OR_: 1.162 (1.130, 1.196), *p* < 0.001], and doctoral education [R_OR_: 1.152 (1.120, 1.185), *p* < 0.001] demonstrate a higher risk of being unaware of proactive health compared to those with a college education or lower; however, this relationship also disappears in Model 2. In Model 2, individuals with primary professional titles [A_OR_: 0.967 (0.937, 0.997), *p* = 0.032] exhibit a lower risk of being unaware of proactive health compared to those without professional titles. This relationship was not observed in Model 1. Conversely, individuals with intermediate professional titles display a higher risk of being unaware of proactive health compared to those without professional titles [R_OR_: 1.077 (1.047, 1.108), *p* < 0.001]. Furthermore, in Model 1, individuals associated with senior professional titles [ROR: 0.879 (0.848, 0.910), *p* < 0.001] and those with full senior professional titles [R_OR_: 0.613 (0.562, 0.669), *p* < 0.001] exhibit a lower risk of being unaware of proactive health compared to those without professional titles. Similar to previous relationships, this effect disappears in Model 2. In both Model 1 and Model 2, individuals with general health status [Model 1 R_OR_: 1.616 (1.580, 1.653), *p* < 0.001; Model 2 A_OR_: 2.351 (2.199, 2.513), *p* < 0.001]5 and those with marginal health status [Model 1 R_OR_: 1.058 (1.031, 1.086), *p* < 0.001; Model 2 A_OR_: 1.200 (1.114, 1.293), *p* < 0.001] exhibit a higher risk of being unaware of proactive health compared to individuals with good health status. Healthcare workers who have not had experience in science popularization [A_OR_: 1.735 (1.674, 1.799), *p* < 0.001], those who have not participated in science popularization activities [A_OR_: 2.260 (2.210, 2.311), *p* < 0.001], and members of the science popularization expert database outside their medical institution [A_OR_: 3.143 (3.023, 3.267), *p* < 0.001] are more likely to be aware of proactive health. Compared to healthcare workers who perceive the current medical relationships as harmonious, those who regard them as average [A_OR_: 1.704 (1.664, 1.744), *p* < 0.001] or severe [A_OR_: 2.163 (2.075, 2.254), *p* < 0.001] are more likely to be aware of proactive health. Additionally, compared to healthcare institution staff with average awareness of health actions and general willingness for science popularization, those with low awareness of health actions and low willingness for science popularization [A_OR_: 1.568 (1.508, 1.631), *p* < 0.001], as well as those with low awareness of health actions but high willingness for science popularization [A_OR_: 1.503 (1.024, 1.082), *p* < 0.001], are more likely to be aware of proactive health. Conversely, healthcare institution staff exhibiting high awareness of health actions and high willingness for science popularization are less likely to be aware of proactive health [A_OR_: 0.370 (0.359, 0.380), *p* < 0.001].

**Table 3:** Binary logistic regression results.

Variable	Model 1 (Unadjusted)		Model 2 (Adjust)	
	OR (95% CI)	*p*-value	OR (95% CI)	*p*-value
Nation				
Han	Ref.	Ref.	Ref.	Ref.
Zhuang	1.014 (0.994, 1.035)	0.17	0.993 (0.970, 1.016)	0.535
Other	1.103 (1.064, 1.143)	<0.001	1.037 (0.996, 1.080)	0.077
Marriage				
Unmarried	Ref.	Ref.	Ref.	Ref.
Married	0.910 (0.890, 0.930)	<0.001	1.020 (0.988, 1.052)	0.219
Other	0.908 (0.857, 0.961)	<0.001	0.981 (0.916, 1.051)	0.586
Age (years)				
18–35	Ref.	Ref.	Ref.	Ref.
36–60	0.937 (0.920, 0.955)	<0.001	1.079 (1.042, 1.117)	<0.001
> 60	0.748 (0.631, 0.887)	<0.001	1.030 (0.844, 1.256)	0.773
Work experience (years)				
≤5	Ref.	Ref.	Ref.	Ref.
6–10	0.980 (0.955, 1.006)	0.135	0.989 (0.956, 1.023)	0.512
11–20	0.972 (0.947, 0.997)	0.029	0.980 (0.939, 1.023)	0.358
> 20	0.843 (0.820, 0.868)	<0.001	0.908 (0.861, 0.957)	<0.001
Type of institution				
Hospital	Ref.	Ref.	Ref.	Ref.
Primary healthcare institution	0.970 (0.947, 0.993)	0.01	1.004 (0.977, 1.032)	0.782
Specialized public health institution	0.908 (0.856, 0.962)	<0.001	1.001 (0.937, 1.069)	0.975
Education				
Associate degree or below	Ref.	Ref.	Ref.	Ref.
Bachelor’s degree	1.186 (1.152, 1.220)	<0.001	0.977 (0.953, 1.002)	0.074
Master’s degree	1.162 (1.130, 1.196)	<0.001	1.029 (0.971, 1.090)	0.338
Doctoral degree	1.152 (1.120, 1.185)	<0.001	0.936 (0.781, 1.122)	0.474
Professional title				
No title	Ref.	Ref.	Ref.	Ref.
Junior title	1.010 (0.984, 1.037)	0.436	0.967 (0.937, 0.997)	0.032
Intermediate title	1.077 (1.047, 1.108)	<0.001	0.973 (0.937, 1.011)	0.165
Associate senior title	0.879 (0.848, 0.910)	<0.001	0.954 (0.906, 1.004)	0.069
Senior title	0.613 (0.562, 0.669)	<0.001	0.979 (0.878, 1.901)	0.698
Health status				
Good	Ref.	Ref.	Ref.	Ref.
Average	1.616 (1.580, 1.653)	<0.001	1.058 (1.031, 1.086)	<0.001
Below average	2.351 (2.199, 2.513)	<0.001	1.200 (1.114, 1.293)	<0.001
Popular science publications				
Yes	Ref.	Ref.	Ref.	Ref.
No	0.274 (0.266, 0.283)	<0.001	1.735 (1.674, 1.799)	<0.001
Health education and promotion activities				
Yes	Ref.	Ref.	Ref.	Ref.
No	3.420 (3.353, 3.489)	<0.001	2.260 (2.210, 2.311)	<0.001
Science popularization experts				
Yes	Ref.	Ref.	Ref.	Ref.
No	5.377 (5.190, 5.571)	<0.001	3.143 (3.023, 3.267)	<0.001
Doctor-patient relationship				
Harmonious	Ref.	Ref.	Ref.	Ref.
Moderate	2.706 (2.651, 2.763)	<0.001	1.704 (1.664, 1.744)	<0.001
Conflictual	3.980 (3.836, 4.129)	<0.001	2.163 (2.075, 2.254)	<0.001
Classification				
Class 1	Ref.	Ref.	Ref.	Ref.
Class 2	2.706 (2.651, 2.763)	<0.001	1.568 (1.508, 1.631)	<0.001
Class 3	3.980 (3.836, 4.129)	<0.001	1.503 (1.024, 1.082)	<0.001
Class 4	0.252 (0.245, 0.259)	<0.001	0.370 (0.359, 0.380)	<0.001

### 3.5 Mediation effects

**Figure 2:**
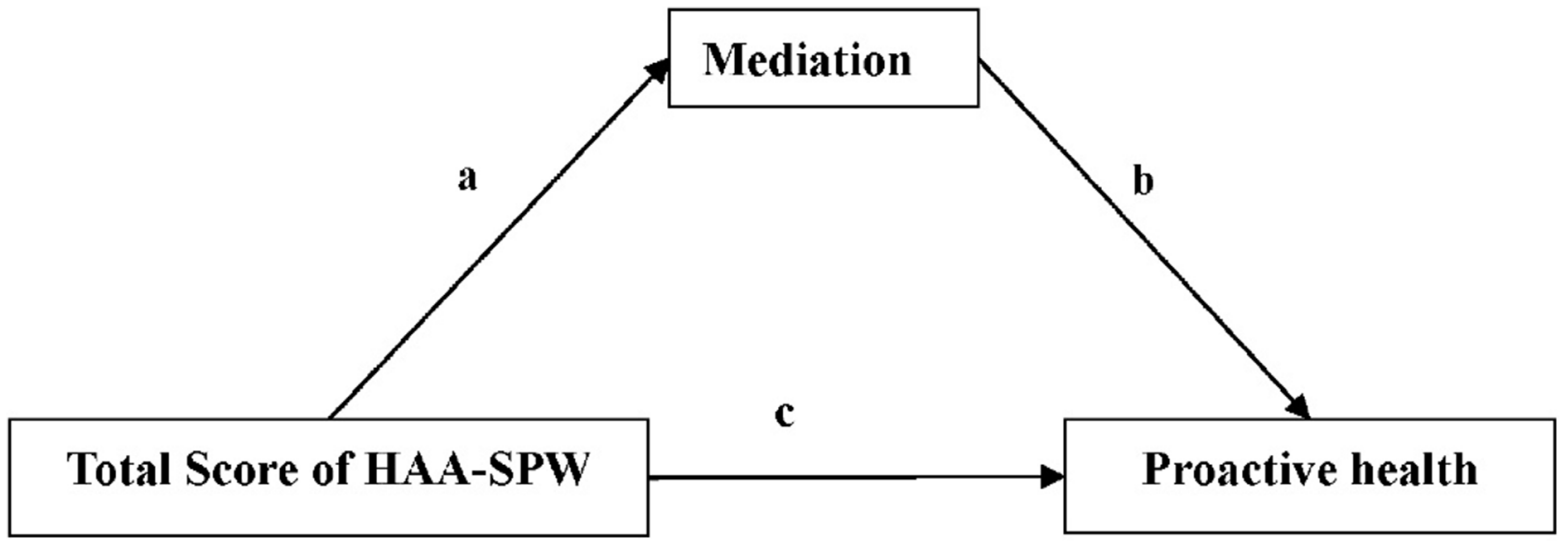
Mediator effect results. Result: a=0.033***; b = −0.037***; c = −0.022***; Mediation = −0.0007 (−0.0008, −0.0006)***; Total effect = −0.0130 (−0.0131, −0.0129)***; Proportion Mediated = 5.12%. Mediation, The level of satisfaction with the current health action measures taken by the government; HAA-SPW, Health Action Awareness and Science Popularization Willingness; ***, *p* < 0.001.

As illustrated in Figure 2, the analysis of mediation effects indicates that the scores of healthcare institution personnel regarding awareness of health actions and willingness to engage in science popularization indirectly influence their awareness of ‘proactive health’ through satisfaction with government health action measures. Specifically, as the scores for awareness of health actions and willingness to engage in science popularization increase, the satisfaction of healthcare institution personnel with the health action measures implemented by the government significantly improves (*a* = 0.033, *p* < 0.001). Further analysis reveals that higher satisfaction is associated with a lower risk of not having heard of ‘proactive health’ (*b* = −0.037, *p* < 0.001). Even after controlling over satisfaction as a mediating variable, the increase in scores for awareness of health actions and willingness to engage in science popularization remains significantly associated with a decrease in awareness of ‘proactive health’ (*c* = −0.022, *p* < 0.001). Through the mediating effect of satisfaction with government health action measures, the indirect impact of awareness of health actions and willingness to engage in science popularization on awareness of ‘proactive health’ is significant, accounting for 5.12% of the total effect.

## 4 Discussion

In this study, 55.54% of healthcare workers indicated that they had heard of the concept of “active health.” However, due to the limitations of the research tools, the depth of their understanding of this concept could not be further explored. To gain a more comprehensive understanding of healthcare workers’ knowledge, the research team will need to develop more advanced assessment tools for evaluating their familiarity with the concept of “active health.” Regardless of gender, healthcare professionals display a similar level of proactive health awareness, indicating that gender does not have a significant impact on the cognition of proactive health. This could be resulted from shared professional environments and information access channels, as well as the training and educational mechanisms within healthcare institutions, which contributes to reducing cognitive differences between genders. Different ethnicities, marital statuses, ages, work experiences, types of employing institutions, educational levels, and professional titles significantly impacted on healthcare personnel’s awareness of “proactive health.” After adjusting for covariates, this relationship ceased to be significant, proving influence of these factors may be indirect or mediated by other variables. Even after adjustment, the health status of healthcare personnel still significantly influences their awareness of ‘proactive health.’ Research indicates that health status is closely related to health literacy, and ‘proactive health’, as an emerging health model, is a concrete manifestation of health literacy (22).

Previous studies suggest that experiences in health education or health communication can enhance an individual’s mastery of comprehensive health knowledge (23). However, this study found that after adjusting for confounding factors, healthcare professionals or health expert pool members with health communication experience were less likely to have heard of proactive health than those without such experience. The reason for this shift may be that the controlled covariates—such as education level and years of work experience—affect their information screening criteria, given that proactive health is an emerging concept. Compared to healthcare personnel who perceive the doctor-patient relationship as harmonious, those who view it as average or poor are more likely to have heard of proactive health. This suggests that, in a tense doctor-patient environment, staff members are more focused on proactive health information to enhance patients’ health management capabilities and satisfaction.

The health action cognition and health education willingness of healthcare personnel in Guangxi are categorized into four subgroups: moderate health cognition and general willingness for health education, low health action cognition and low willingness for health education, low health action cognition and high willingness for health education, and high health action cognition and high willingness for health education. This classification reveals significant heterogeneity within this group, indicating considerable individual differences in health action cognition and willingness for health education. It reflects the diversity in health cognition and educational willingness among healthcare personnel in Guangxi. Individuals exhibiting low health action cognition and educational willingness often exhibit a significant demand for health information. Consequently, they are more likely to seek out and engage with emerging health concepts, such as ‘proactive health,’ to address their cognitive deficiencies (24). The department is advised to implement targeted incentive mechanisms for healthcare personnel exhibiting low health-action cognition and limited willingness to disseminate health education, so as to sustain and amplify their demand for emerging health concepts and ultimately foster a positive “demand–acquisition–redemand” cycle. Furthermore, individuals with high health action cognition and educational willingness tend to possess a higher level of health literacy and a substantial reserve of health knowledge, which results in a relatively lower demand for emerging concepts (25). This phenomenon of ‘information saturation’ may lead to a decreased likelihood of encountering or paying attention to the concept of ‘proactive health‘(26). Moreover, over 70% of the participants worked in hospitals, where attention is primarily directed toward clinical expertise and specific policies. ‘Proactive health’ being a macro-level strategy may therefore have received less notice among staff who otherwise demonstrate high health-action cognition and strong willingness to engage in health education, potentially explaining their lower reported awareness of the concept. Therefore, for this specific group of healthcare workers, we should target the micro-level clinical issues they encounter daily as entry points. This involves translating the “proactive health” concept into actionable micro-level strategies that directly improve their clinical decision-making and departmental management, thereby achieving a cognitive shift from macro-level concepts to micro-level practice.

The mediation effect analysis indicates that health action cognition and willingness to disseminate health information among healthcare personnel indirectly influence their understanding of “proactive health” through satisfaction with government health initiatives. Higher levels of health action cognition and willingness to engage in health education may enhance recognition and trust in government health measures, thereby facilitating the acceptance of emerging health concepts (27). However, an increase in health action cognition and willingness to engage in health education is significantly associated with a decrease in understanding of “proactive health.” This finding aligns with individual-centered binary logistic regression analysis, which shows that groups with high health action cognition and willingness to disseminate health information are at greater risk of being unaware of the concept of “proactive health.” This may be because, despite healthcare personnel’s relatively high levels of health-action cognition and willingness to engage in health education, cognitive barriers or difficulties in integrating new information can still arise when they are confronted with novel concepts. This could hinder their ability to simultaneously enhance their understanding of “proactive health” (28). Analysis of the mediating effects of satisfaction with government health measures reveals that health action cognition and willingness to disseminate health information have a significant indirect impact on understanding “proactive health,” accounting for 5.12% of the total effect. This result suggests that, although satisfaction plays a mediating role, its explanatory power is limited, indicating a need to incorporate additional variables not captured in the present study. Despite the 5.12% effect being statistically small, it translates into an absolute gain of roughly 26,000 individuals among Guangxi’s more than 510,000 healthcare workers and thus carries potential public-health value. Policymakers considering raising satisfaction with government health initiatives to enhance workers’ health cognition and dissemination willingness must therefore weigh intervention costs against the resulting cognitive benefits. Given that Guangxi, along with the minority autonomous regions of Xinjiang, Ningxia, and Inner Mongolia, is situated in western or border areas where grassroots medical resources are comparatively scarce, and all operate under the unified national “Healthy China Initiative,” their institutional environments and policy contexts are highly similar. Consequently, the present findings provide a robust foundation for subsequent comparative studies across these regions.

This research constitutes a large-scale cross-sectional survey that encompasses nearly one-third of healthcare professionals in the Guangxi Zhuang Autonomous Region, aimed to assess their level of awareness regarding the concept of ‘proactive health.’ Furthermore, this study utilizes latent class analysis to investigate the heterogeneity among healthcare professionals in Guangxi regarding their understanding of health-related actions and their demand for science popularization, thereby enabling a more accurate identification of the factors influencing their awareness of proactive health. However, this study has several limitations. Firstly, due to the cross-sectional design, this study cannot establish causal relationships. Secondly, the study sample is drawn exclusively from the Guangxi Zhuang Autonomous Region in southern China, which may be influenced by local environmental, cultural, and socio-economic factors. As a result, the generalizability of the conclusions may be somewhat limited. Additionally, due to the geographical constraints of data collection, the findings may not accurately reflect the perspectives of healthcare professionals from other regions or cultural backgrounds across the country. The questionnaire in this study serves as an exploratory tool to collect baseline data on healthcare professionals’ perceptions. It has not been formally validated using statistical methods, and only the Cronbach’s alpha coefficient test was conducted. To enhance its quality, the research team undertook the following steps: (a) reviewing relevant health policy evaluation literature; (b) consulting public health experts; and (c) conducting a pre-survey with healthcare professionals to refine the phrasing of the questions.

## 5 Conclusion

In this study, significant group heterogeneity was observed among healthcare professionals in Guangxi regarding their understanding of health-related actions and their willingness to engage in science popularization. This difference notably influences their awareness of the concept of ‘proactive health.’ When implementing new health policies, relevant authorities should focus on those professionals who demonstrate a high level of understanding of health-related actions and a strong willingness to engage in science popularization. Furthermore, enhancing healthcare professionals’ satisfaction with government health initiatives are essential, as this will create favorable conditions for further promoting public health.

## Data Availability

The raw data supporting the conclusions of this article will be made available by the authors, without undue reservation.

## Footnotes

^1^ https://www.wjx.cn/
